# Schrödinger’s T Cells: Molecular Insights Into Stemness and Exhaustion

**DOI:** 10.3389/fimmu.2021.725618

**Published:** 2021-08-26

**Authors:** Nancy M. Gonzalez, Dawei Zou, Andy Gu, Wenhao Chen

**Affiliations:** ^1^Immunobiology & Transplant Science Center, Department of Surgery, Houston Methodist Research Institute & Institute for Academic Medicine, Houston Methodist Hospital, Houston, TX, United States; ^2^College of Medicine, Texas A&M Health Science Center, College Station, TX, United States; ^3^Department of Surgery, Weill Cornell Medicine, Cornell University, New York, NY, United States

**Keywords:** T cell, T cell exhaustion, T cell stemness, transplantation, transcription factor, epigenetic regulation

## Abstract

T cell stemness and exhaustion coexist as two key contrasting phenomena during chronic antigen stimulation, such as infection, transplant, cancer, and autoimmunity. T cell exhaustion refers to the progressive loss of effector function caused by chronic antigen exposure. Exhausted T (T_EX_) cells highly express multiple inhibitory receptors and exhibit severe defects in cell proliferation and cytokine production. The term T cell stemness describes the stem cell-like behaviors of T cells, including self-renewal, multipotency, and functional persistence. It is well accepted that naïve and some memory T cell subsets have stem cell-like properties. When investigating the exhaustive differentiation of T cells in chronic infection and cancer, recent studies highlighted the stemness of “precursors of exhausted” T (T_PEX_) cells prior to their terminal differentiation to T_EX_ cells. Clinically successful checkpoint blockades for cancer treatment appear to invigorate antitumor T_PEX_ cells but not T_EX_ cells. Here we discuss the transcriptional and epigenetic regulations of T cell stemness and exhaustion, with a focus on how systems immunology was and will be utilized to define the molecular basis underlying the transition of T_PEX_ to T_EX_ cells. We suggest a “stepwise model” of T cell stemness and exhaustion, in which loss of stemness and exhaustion progression are gradual multi-step processes. We provide perspectives on the research needed to define T cell stemness and exhaustion in the transplantation setting, in which allogenic T cells are also chronically exposed to alloantigens. A better understanding of T cell stemness and exhaustion will shed light on developing novel strategies for immunotherapies.

## Introduction

The adaptive immune system confers an evolutionary advantage to vertebrates with its highly tailored defense against a myriad of environmental pathogens. During acute infections, naïve T cells of the adaptive immune system are activated in an antigen-specific manner and expand into large numbers of effector T cells. Following the clearance of pathogens, most activated T cells die, but a small portion develop into memory T cells. Naïve and memory T cells behave somewhat like stem cells. Naïve and memory T cells for a given antigen are capable of self-renewing in the absence of antigen, persist in low abundance in the body, and replenish functional effector cells upon antigen (re-)encounter. The term “T cell stemness” is used to describe stem cell-like behaviors of naïve and memory T cells (1, 2).

Chronic infections occur when the host immune system fails to clear the pathogens. In this context, antigen-specific T cells gradually develop into a dysfunctional cell state termed T cell exhaustion. Exhausted T (T_EX_) cells express high levels of inhibitory receptors (e.g., PD-1, TIM-3, LAG-3, TIGIT), progressively lose cytokine production (e.g., IFN-γ, TNF-α) and proliferative capacity, and have a very limited ability to control infections. Recent advances in systems immunology have opened a new avenue for defining T cell states in diseases. Single-cell sequencing technologies have often identified T_EX_ cells in various kinds of solid tumors. T cell exhaustion has thus been recognized as an underlying principle of T cell dysfunction in both chronic infection and cancer (3).

The PD-L1–PD-1 checkpoint blockade has now become a pillar of cancer therapy. PD-L1–PD-1 blockade was thought to promote antitumor immunity by reversing exhaustion of PD-1^+^ T cells. Using systems immunology to study how PD-L1–PD-1 blockade works, researchers found that terminally differentiated TCF1^−^PD-1^+^ T_EX_ cells represent an irreversible T cell state, and that PD-L1–PD-1 blockade promotes antitumor immunity mainly by invigorating TCF1^+^PD-1^+^ “precursors of exhausted” T cells (T_PEX_) (3). We now know that T_PEX_ cells have memory-like properties and maintain aspects of T cell stemness. T_PEX_ cells not only continuously generate T_EX_ cells during chronic antigen exposure, but also give rise to functional T cells and sustain chronic T cell response even in the absence of checkpoint blockade. Hence, T cell stemness and exhaustion, two seemly contrasting phenomena, coexist during chronic antigen stimulation.

In this review article, we present an overview of characteristics and the molecular basis of T cell stemness and exhaustion. We provide a fundamental concept that T cell stemness and exhaustion together determine the magnitude of chronic T cell response. Currently, T cell stemness and exhaustion are far less studied in the transplantation setting compared with those in chronic infection and cancer research. We highlight this research gap for discussion. Systems immunology approaches are timely and highly relevant to characterizing how T cell stemness and exhaustion together determine transplant outcomes.

## Stemness of Memory T Cells

Adaptive immunity hinges on creating immunological memory after natural acute infection or vaccination. Generating long-lived antigen-specific memory lymphocytes forms the cellular basis of immunological memory, which has been extensively studied and reviewed (4, 5). Here we discuss the concept of T cell stemness as a basic mechanism that maintains T cell memory. T cell stemness encompasses the capacity of a T cell to self-renew as well as to differentiate into multiple downstream cell types, an ability called multipotency.

Early studies examining memory T cells in mice identified CD62L^+^CD44^+^ central memory T (T_CM_) and CD62L^–^CD44^+^ effector memory T (T_EM_) cells. T_CM_ cells are less terminally differentiated than T_EM_ cells. Graef et al. have investigated the self-renewal capacity and multipotency of individual CD8^+^ T_CM_ cells. In their experimental approach, only one single antigen-specific naïve T or T_CM_ cell was adoptively transferred to each host. They found that the transferred single naïve T cells and single T_CM_ cells produced remarkably similar progeny size and diversity in response to antigen stimulation. Moreover, progeny derived from single primary T_CM_ cells contained secondary T_CM_ cells. When secondary T_CM_ cells were individually transferred to new hosts and exposed to antigen, they again generated a diverse offspring cells, including effector T (T_EFF_), T_CM_, and T_EM_ cells (2). This study elegantly revealed the stemness of naïve and T_CM_ cells.

There is a naive-like memory T cell population even less differentiated than T_CM_ cells. These cells were designated memory stem T cells (T_SCM_) to reflect their enhanced capacity for self-renewal and multipotent capacity to differentiate into all memory and effector T cell subsets (6–8). T_SCM_ cells bear many similarities to naive T cells, such as CD62L^+^CD44^–^ in mice and CD45RO^–^CD45RA^+^CD62L^+^IL-7Rα^+^ in human. However, they are generated after antigen stimulation and express elevated levels of Bcl-2, Sca-1, IL-2Rβ, CXCR3, and CD95 (6, 9).

T_SCM_ cells develop directly from naïve T cells. Cieri et al. discovered that IL-7 is critical for the development of human T_SCM_ cells from naïve T cells *ex vivo*, whereas IL-15 supports their expansion. IL-7/IL-15−instructed and gene-modified T_SCM_ cells surpass other memory T cell subsets for the ability to expand and differentiate into T_EFF_ cells, as well as to mediate severe xenogeneic GVHD upon adoptive transfer into immunodeficient mice (10). These findings not only reveal the biologic requirements for T_SCM_ cell development and expansion from naïve T cells, but also highlight the optimal cytokine condition for differentiation and expansion of genetically engineered T_SCM_ cells suitable for cancer adoptive cellular therapy.

Roberto et al. studied T_SCM_ cell generation in preclinical models of hematopoietic stem cell transplantation (HSCT). T_SCM_ cells arise from donor naïve T cells and are abundant early after haploidentical HSCT combined with posttransplant cyclophosphamide therapy. T_SCM_ cells exhibit superior reconstitution capacity (11). Cieri et al. investigated T_SCM_ cell generation in patients undergoing haploidentical HSCT. T_SCM_ cells can differentiate directly from naïve precursors that are adoptively transferred with the graft. Similar to naïve T cells, T_SCM_ cells are able to generate the complete spectrum of T cell memory, including T_SCM_ cells. By contrast, few T_CM_ and T_EM_ cells can convert into T_SCM_ cells (12). Likewise, Oliveira et al. traced the thymidine kinase (TK) transduced T cells infused in patients after haploidentical HSCT. The phenotypic distribution of cytomegalovirus (CMV)- and Flu-specific TK^+^ T cells in patients was investigated. Only when antigen-specific T_SCM_ cells are detected in the infused cell population, a complete spectrum of antigen-specific T_SCM_, T_CM_, and T_EM_ can be retrieved long-term in patients (13). These studies support a progressive model of memory T cell differentiation, following naïve T → T_SCM_ → T_CM_ → T_EM_. TK transduction of T cells prior to infusion has a unique advantage for tracing the infused TK^+^ T cells overtime in patients after haploidentical HSCT. At a median follow-up of 6.8 years after cell infusion, low but stable levels of TK^+^ T_SCM_, T_CM_, and T_EM/EFF_ cells are detected in all patients. Of note, the absolute counts of long-term persisting TK^+^ cells are positively correlated with the amount of infused T_SCM_ cells (13). This finding highlights that T_SCM_ cells govern the quality and longevity of the adoptively transferred T cells. Consistent with this report, Biasco et al. analyzed T_SCM_ cells in adenosine deaminase (ADA) deficient patients after infusion of ADA transduced mature lymphocytes or hematopoietic stem cells. Gene-transduced T_SCM_ cells persist and preserve their precursor potential in patients for up to 12 years after infusion (14). Fuertes Marraco et al. tracked yellow fever (YF) specific CD8^+^ T cells in humans after YF vaccination. Frequencies of YF-specific CD8^+^ T_CM_ and T_EM_ cells decrease with time, but naïve-like YF-specific CD8^+^ T_SCM_ cells are stably maintained for more than 25 years (15). Taken together, these studies show the direct development of T_SCM_ cells from naïve T cells and provide fundamental insights into the stemness capacities of T_SCM_ cells. The self-renewal capacity leads to the long-term persistence of T_SCM_ cells, whereas multipotency enables T_SCM_ cells to differentiate into the complete spectrum of memory and effector cells. The stemness of T_SCM_ cells should be taken into consideration when developing vaccines or immunotherapies.

Wnt–β-catenin, an evolutionarily conserved pathway, plays an essential role in regulating cell stemness. Gattinoni et al. discovered that Wnt signaling also promotes stemness in memory T cells (7). The Wnt pathway effector TCF1 has now been recognized as a central player of T cell stemness, which will be discussed throughout this review (16).

## T Cell Exhaustion During Chronic Antigen Exposure

The seeming antithesis of “stemness” and self-renewal is “exhaustion”. The broad term describes a T cell state first seen in chronic viral infection (17, 18). Almost three decades ago, Moskophidis et al. infected mice with the noncytopathic lymphocytic choriomeningitis virus (LCMV) strain Docile (D). The presence of large numbers of LCMV-D produced high antigen loads that forced exhaustion of the specific antiviral CD8^+^ cytotoxic T cells. Due to the technical limitation at that time, the authors indicated that their findings explained mechanistically, but not yet molecularly, the puzzle of why high-dose LCMV infection diminishes T cell responses (18, 19).

Nowadays, systems immunology approaches permit insight beyond the traditional cell surface-marker or cytokine expression, allowing us to peer at the transcriptional, epigenetic, and metabolic programming underpinning various T cell states. In 2007, Wherry et al. used microarray analysis to investigate the molecular signature of CD8^+^ T cell exhaustion after chronic LCMV infection. CD8^+^ T_EX_ cells overexpressed PD-1 and other inhibitory receptors, expressed a distinct set of transcription factors, and had profound metabolic and bioenergetic deficiencies (20). These findings initiatively revealed how T_EX_ cells progressively lose effector functions at the molecular level.

ScRNA-seq technique has opened a new dimension for studying T cell biology. Many of the initial scRNA-seq studies examined T cell states in different types of cancer. CD8^+^ T_EX_ cells are often preferentially enriched in the tumor microenvironment (21, 22). Since then, T cell exhaustion has become an accepted term to describe the response of T cells to both chronic infection and cancer (23–29).

## Stemness of T_PEX_ Cells

PD-1–PD-L1 checkpoint blockade is effective for treatment of different cancers. Because T_EX_ cells often express high levels of PD-1, reversal of T cell exhaustion was considered as a mechanism underlying PD-1–PD-L1 blockade. This view was dramatically changed in 2016 when TCF1^+^PD-1^+^ T_PEX_ cells were discovered. Im et al. investigated how PD-1 blockade regulates CD8^+^ T cell responses during chronic LCMV infection. They found that a TCF1^+^PD-1^+^ CD8^+^ T-cell subset resembles stem cells during chronic LCMV infection, undergoing self-renewal and also differentiating into terminal TCF1^–^PD-1^+^ T_EX_ cells. TCF1 is not only a marker for this cell subset, but also has an important role in their generation. More importantly, the proliferative burst after PD-1 blockade comes almost exclusively from this cell subset (30). This cell subset has amassed many names, including “stem-like”, “memory-like”, “follicular cytotoxic”, “exhausted progenitor”, and “precursors of exhausted” T cells. Herein we prefer the term “precursors of exhausted” T (T_PEX_) that better describes the relationship between T_PEX_ and T_EX_ cells (3).

Siddiqui et al. have investigated the stem-like properties of TCF1^+^PD-1^+^ tumor-infiltrating CD8^+^ T lymphocytes (TILs). TCF1^+^PD-1^+^ TILs exhibited stem-like functions as they mediated the proliferative response to immunotherapy, generating both TCF1^+^PD-1^+^ and differentiated TCF1^–^PD-1^+^ cells. A *Tcf7*-diphtheria toxin receptor (DTR) system was creatively designed to deplete tumor-specific TCF1^+^CD8^+^ T cells. Ablation of TCF1^+^PD-1^+^ TILs indeed restricted responses to immunotherapy. The authors concluded that immune checkpoint blockade relies on the proliferation of stem-like TCF1^+^PD-1^+^ TILs, but not on reversal of T cell exhaustion programs (31).

In a viewpoint article, 18 experts in the T cell biology field described their thoughts on T cell exhaustion. They highlighted that terminally differentiated TCF1^–^ T_EX_ cells are derived from the self-renewing TCF1^+^ precursor population, with stem cell-like properties akin to memory T cell populations (29). The term “stemness” is now increasingly being used in the literature to refer to the stem cell-like properties of T_PEX_ cells.

## Transcriptional Regulation of T Cell Stemness

Fearon et al. postulated twenty years ago that the stem cell-like capacity not only promotes T cell memory but also sustains chronic T cell response, and that some transcription factors may exist in T cells to maintain stem cell-like capacity and restrain terminal differentiation (32). Since then, transcription factors regulating T cell stemness have been gradually revealed. The transcription factor TCF1 has been identified as not only a marker, but also a key regulator of memory T cell stemness. During an acute infection, antigen-specific CD8^+^ T cells can be broadly divided into IL-7Rα^low^KLRG1^hi^ terminal effectors and IL-7Rα^hi^KLRG1^low^ memory precursors. TCF1-deficient mice mount a normal primary effector T cell response, but they essentially lack CD8^+^ memory precursors at the peak of acute infection (33). After acute infection, TCF1 deficiency severely impairs CD8^+^ T_CM_ differentiation, longevity, and response to recall antigens (33, 34). These findings demonstrate that TCF1 programs CD8^+^ memory T cell stemness and fate.

TCF1 is also both a marker and a key regulator for T_PEX_ cells during chronic antigen exposure (30, 35). TCF1 deficiency abrogates T_PEX_ cell generation, leading to an impairment of viral control (30, 35). Chen et al. used scRNA-seq and lineage tracing to study T cell states early during chronic infection. They found that TCF1 represses the development of KLRG1^hi^ terminal effector T cells while fostering T_PEX_ cells (36). Hence, although TCF1 promotes T_PEX_ cell generation and sustains chronic response, it may restrain T cells to exert immediate effector function (36).

The transcription factor BACH2 is required for CD8^+^ T cell memory differentiation. Roychoudhuri et al. mapped genome-wide BACH2 binding sites using ChIP-seq. They found that BACH2 binds to enhancers of TCR-driven genes, attenuating the availability of AP-1 sites to Jun family transcription factors. This process restrains terminal effector programs. Effector T cells thus downregulate BACH2 expression to promote effector fate differentiation. By contrast, BACH2 expression enables the generation of long-lived memory cells (37).

BACH2 is an essential regulator for T_PEX_ cells during chronic antigen exposure. Utzschneider et al. and Yao et al. independently showed that BACH2 deficiency impairs the generation of TCF1^+^ CD8^+^ T_PEX_ cells, whereas BACH2 overexpression enforces their generation at the early phase of chronic infection (38, 39). By using single-cell transcriptomic and epigenomic approaches, Yao et al. further revealed that BACH2 itself is transcriptionally and epigenetically active in T_PEX_ cells but not in T_EX_ cells. Moreover, BACH2 establishes the transcriptional and epigenetic programs of stem-like CD8^+^ T_PEX_ cells, while suppressing the molecular program that drives terminal exhaustion. Therefore, BACH2 actively enforces commitment to stem-like CD8^+^ T_PEX_ lineage and guards T_PEX_ cells against terminal exhaustion (39).

Id3, a member of the Id protein family which negatively regulates E-protein DNA-binding (40), is essential for CD8^+^ memory T cell formation (41). In the above-mentioned study by Utzschneider et al., Id3 was highly expressed among TCF1^+^TIM3^–^ T_PEX_ cells. Both Id3 and TCF1 are progressively lost when T_PEX_ cells terminally differentiate into TIM3^+^ T cells (38). Thus, both Id3 and TCF1 are transcriptional markers of T_PEX_ cells. Whether Id3 regulates the T_PEX_ cell state remains unexamined.

The transcription factor c-Myb is another pivotal regulator of CD8^+^ T cell stemness. Following an antitumor vaccination, *Myb*-deficient CD8^+^ T cells are prone to terminal differentiation, generating fewer stem cell-like Tcm cells than do *Myb*-sufficient T cells. By contrast, c-Myb overexpression promotes CD8^+^ T cell memory and recall responses that elicit therapeutic antitumor immunity. Mechanistically, c-Myb acts as a transcriptional activator of *Tcf7* (encoding TCF1) to enhance stemness and as a repressor of *Zeb2* to restrain effector differentiation (42). Systems immunology approaches have further shown that in CD8^+^ T cells, c-Myb promotes formation of T_SCM_ cells, restrains terminal differentiation, and fosters TCF1^+^ T_PEX_ cell persistence (36, 43).

**Figure 1:**
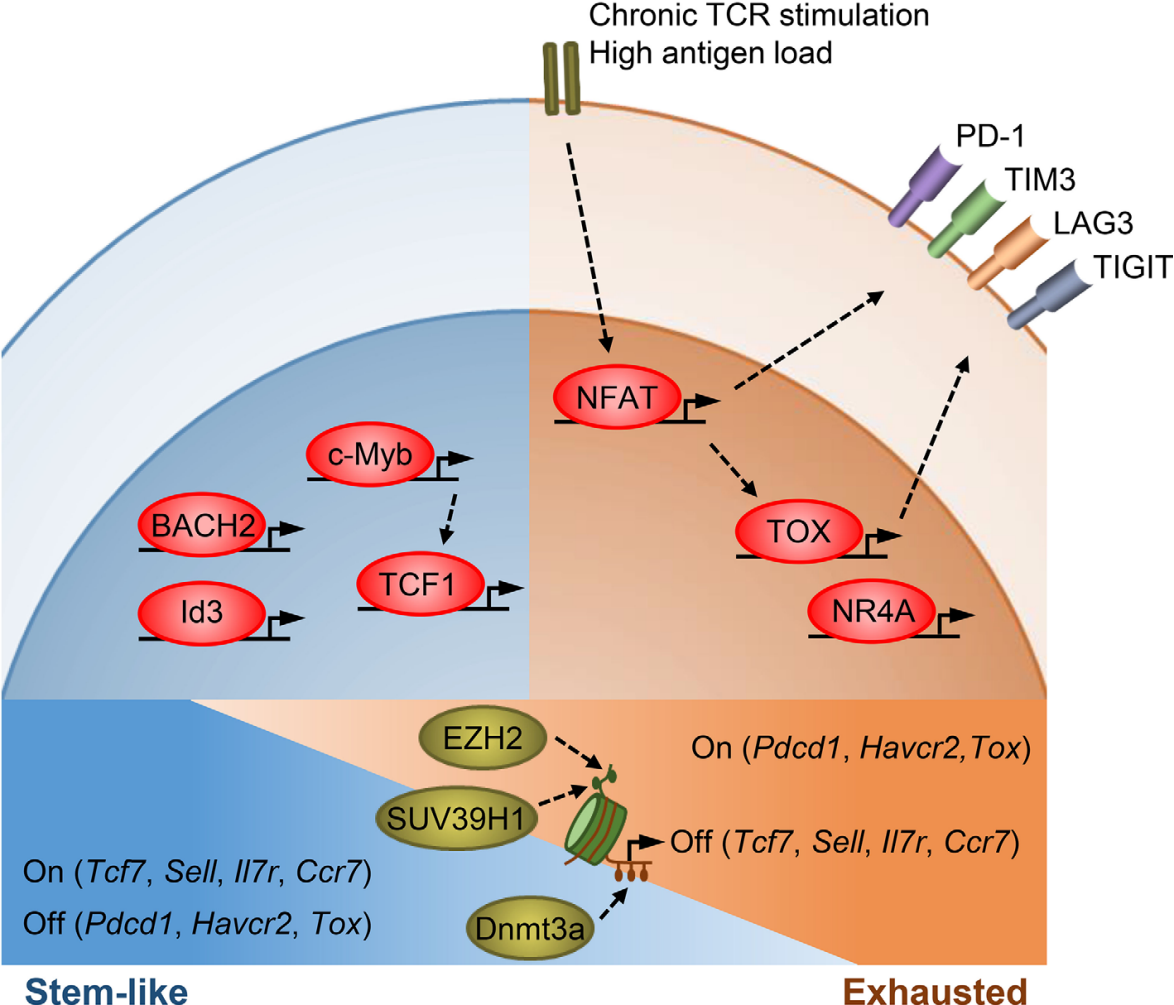
Transcriptional and epigenetic regulation of T cell stemness and exhaustion. Transcription factors TCF1, BACH2, Id3, and c-Myb promote memory T cell formation as well as stemness in T_PEX_ cells. TCF1, BACH2, and c-Myb also restrain effector T cell differentiation. c-Myb is a transcriptional activator of *Tcf7*. Under chronic antigen exposure or high antigen load, the TCR-NFAT-TOX/NR4A axis drives the exhaustion program of T cells and promotes the expression of multiple inhibitory receptors. TOX/TOX2 and NR4As are secondary transcription factors induced by initiating NFAT. Epigenetic mechanisms, such as histone modifications and DNA methylation, act in concert with transcription factors to regulate T cell states. SUV39H1 and EZH2 silence stem/memory genes during effector T cell differentiation by methylating H3K9 and H3K27, respectively. Dnmt3a mediates *de novo* DNA methylation to inhibit the expression of stem/memory genes during effector differentiation. T_EX_ cells have an epigenetic profile distinct from that of T_EFF_ and memory T cells, though how epigenetic enzymes regulate the expression of exhaustion genes remains unclear.

Above discoveries suggest that memory cell formation and T_PEX_ cell generation are controlled by the same set of transcription factors, which maintains cell stemness and restrains terminal differentiation (**Figure 1**).

## Transcriptional Regulation of Exhaustion

T cell exhaustion occurs during chronic antigen stimulation. We and others showed that high antigen load drives T cell exhaustion. High antigen load not only maintains antigen chronicity but may also provide repeated antigen stimulation *via* TCR to initiate exhaustive differentiation during the early phase of a chronic response (44).

TCR-induced calcium-calcineurin signal activates the NFAT family of transcription factors. Activated NFAT proteins in turn interact with other transcription factors (e.g., AP-1 family) to govern T cell activation and effector cell differentiation. Martinez et al. demonstrated that NFAT proteins also drive the transcriptional program of CD8^+^ T cell exhaustion. CD8^+^ T cells lacking NFAT fail to express exhaustion-related inhibitory receptors. The authors generated an engineered form of NFAT1 that cannot interact with AP-1 transcription factors. With this engineered NFAT, they showed that NFAT drives T cell exhaustion by binding at genomic regions sites that do not require cooperation with AP-1 (45).

Calcineurin inhibitor FK506, a mainstay immuno-suppressant, inhibits NFAT nuclear translocation and its transcriptional activity. Philip et al. showed that low-dose FK506 decreases the expression of inhibitory receptors PD1 and LAG-3 and increases TCF1 expression in CD8^+^ T cells during chronic exposure to a tumor antigen. Hence, partial downregulation of NFAT activity helps to prevent T cell exhaustion/dysfunction (46).

TOX is a secondary transcription factor induced by initiating NFAT. TOX likely does not participate in the differentiation of effector and memory T cell states in response to acute infection. On the contrary, in chronic infection and cancer, TOX is a key driver of the exhaustion program in CD8^+^ T cells. In year 2019, five papers from different groups reported that TOX expression is pivotal for the formation and maintenance of TCF1^+^ T_PEX_ cells and the subsequent differentiation of terminal T_EX_ cells (47–51). Without TOX, the T_PEX_ cell formation is impaired, and T_EX_ cells do not form. *Tox*-deleted CD8^+^ cells did not upregulate genes for inhibitory receptors, such as *Pdcd1*, *Entpd1*, *Havcr2*, *Cd244* and *Tigit* (48).

Although TOX drives the exhaustion program in CD8^+^ T cells, its deletion does not effectively potentiate chronic T cell response. Scott A et al. showed that *Tox*-deleted antitumor CD8^+^ cells remain dysfunctional in tumors. The reduced expression of inhibitory receptors on them does not prevent the loss of effector function (48). Yao et al. evidenced that ectopic expression of TOX even endows terminal CD8^+^ T_EX_ cells with the ability to persist during chronic viral infection (50). Alfei et al. showed that TOX-mutated T cells initially mediate increased effector function but eventually undergo a massive decline in their quantity. This is possibly due to the critical role of TOX in maintaining TCF1^+^ self-renewing T_PEX_ cells (49).

In a model of CNS autoimmunity, Page et al. showed that TOX is required for the persistence of self-reactive CD8^+^ T cells. The authors used systems immunology to characterize the transcriptional and epigenetic landscape of self-reactive CD8^+^ T cells. They found that continuous exposure to CNS self-antigen sustains TOX expression in self-reactive T cells, and that TOX remodels more than 400 genomic regions including the *Tcf7* loci (52). Therefore, along with the line that TOX maintains TCF1^+^ T_PEX_ cells (49), TOX may modulate TCF1 expression. The above discoveries collectively suggest that T cells express TOX to adapt their response to persistent antigen stimulation, and that TOX affects chronic T cell responses in a multi-factorial, context-dependent manner.

NR4As (NR4A1, NR4A2, and NR4A3) are also secondary transcription factors induced by initiating NFAT. CD8^+^ T cells express high levels of NR4As upon chronic viral infections or in tumors. Chen et al. showed that NR4As have an important role in the program of T cell dysfunction/exhaustion. Knocking out all three NR4As in CAR T cells promotes tumor regression and prolongs the survival of tumor-bearing mice (53). Liu et al. demonstrated that NR4A1 is preferentially recruited to AP-1 binding sites and represses effector-gene expression by inhibiting AP-1 function. Deletion of NR4A1 overcomes T cell dysfunction, exaggerates effector function, and enhances immunity against tumor and chronic virus (54).

BATF is a member of the AP-1 family transcription factors. It dimerizes with JunB and acts as a repressor of AP-1 activities in activated T cells. Quigley et al. suggested that BATF is one of the exhaustion drivers. Elevated BATF expression correlates with CD8^+^ T cell exhaustion in response to HIV infection. Silencing BATF in T cells rescues HIV-specific T cell function (55). BATF, BACH2, and NR4A1 all have repressive roles for AP-1 activities. BACH2 suppresses AP-1–dependent effector genes to sustain T_PEX_ cells, whereas BATF and NR4A1 promote terminal exhaustion. Conversely, overexpression of the canonical AP-1 factor c-Jun has been shown to induce exhaustion resistance of antitumor CAR-T cells (56).

Taken together, the TCR-NFAT-TOX/NR4A axis drives the exhaustion program of CD8^+^ T cells (**Figure 1**). Further studies are required to define how T_PEX_ cells repress their effector program to maintain stemness and how T_EX_ cells forfeit their stemness and effector function.

## Epigenetic Regulation of Stemness During Memory T Cell Development

Upon acute infection, T cells undergo significant epigenetic reprograming to fulfill a variety of functions. Epigenetic mechanisms (e.g., DNA methylation and histone modifications), acting in concert with transcription factors, orchestrate CD8^+^ T cell differentiation and memory. This has been excellently reviewed elsewhere recently (57, 58). Here we focus on the epigenetic regulation of stemness in T cells.

The ontogeny of memory T cells after acute infection has been gradually revealed. Early after an acute infection, antigen-specific CD8^+^ T cells can branch into less-differentiated IL-7Rα^hi^KLRG1^low^ memory precursors and more-differentiated IL-7Rα^low^KLRG1^hi^ terminal effectors. This paradigm highlights that fully differentiated terminal effectors lose memory potential. Herndler-Brandstetter et al. have recently identified an IL-7Rα^+^KLRG1^+^ cell population that retains memory potential. IL-7Rα^+^KLRG1^+^ cells represent effector T cells that are less differentiated than the IL-7Rα^–^KLRG1^+^ terminal effectors. These cells receive intermediate amounts of activating signals, downregulate KLRG1 during the contraction phase, and can differentiate into all memory T cell linages (59). Hence, both IL-7Rα^hi^KLRG1^low^ memory precursors and IL-7Rα^+^KLRG1^+^ effector T cells have memory potential.

Investigation into epigenetic changes provides a better understanding of memory T cell development and stemness. DNA methylation generally inhibits gene expression in cells. Youngblood et al. investigated the *Dnmt3a*-dependent *de novo* DNA methylation in CD8^+^ T cells during acute infection. They found that CD8^+^ memory precursors initially acquire *de novo* DNA methylation programs to inhibit the expression of some naïve-associated genes and become demethylated at the loci of effector-associated genes (permitting expression). To develop into long-lived memory T cells, those memory precursors erase *de novo* methylation programs at naïve-associated gene loci to permit their re-expression, while key effector genes remain demethylated. Thus, T cells may lose and re-express some stemness/naïve-associated genes during memory cell development. Fully developed memory T cells express naïve-associated genes and are also poised to exert effector function upon re-infection (60).

Methylation of histone 3 lysines 9 (H3K9) and 27 (H3K27) is highly correlated with transcriptional repression. SUV39H1 is a histone methyltransferase that methylates H3K9. Pace et al. studied the role of SUV39H1 in regulating CD8^+^ T cell response upon infection. They showed that SUV39H1-dependent trimethylation of H3K9 (H3K9me3) controls the expression of a set of stem-related memory genes. *Suv39h1*-deificent CD8^+^ T cells fail to silence stem/memory genes during terminal effector cell differentiation and have increased long-term memory reprogramming capacity (61). Polycomb repressive complex 2 (PRC2) silences gene expression through methylation of H3K27. EZH2 is the catalytic subunit of PRC2. Gary et al. profiled H3K27me3 in CD8^+^ T cells during viral infection. H3K27me3 deposition at numerous stem/memory genes during effector differentiation. *Ezh2*-deficiency impaired terminal effector cell differentiation (62). Therefore, H3K9me3 and H3K27me3 deposition at stem/memory genes drive effector T cells to lose stemness and memory potential.

Developed memory cell subsets display distinct epigenetic landscapes. Durek et al. investigated the genome-wide profiles of DNA methylation in naive, T_CM_, T_EM_, and terminally differentiated memory T (T_EMRA_) cells. They observed a progressive global loss of DNA methylation in the order naïve- T_CM_ - T_EM_ - T_EMRA_. Mean DNA methylation levels for the entire genome drop from 84% in naïve T cells to 67% in T_EMRA_ cells. This study highlights that developed memory T cells contain subsets with different differentiation states (63).

We interpret the above findings by suggesting that regardless of the types and phases of a T cell response, each state always includes less- or more-differentiated T cells. Stem/naïve/memory genes are expressed in less-differentiated T cells to ensure T cell persistence and are epigenetically silenced in terminal effector T cells to fulfill effector function (**Figure 1**).

## Epigenetic Hallmarks of T Cell Exhaustion

The epigenetic profile of exhausted T cells differs markedly from those of effector and memory T cells. Sen et al. used ATAC-seq to define chromatin-accessible regions (ChARs) in CD8^+^ T cells. Upon both acute and chronic infections, antigen stimulation of naïve T cells leads to dramatic remodeling of ChARs. About 71% ChARs either emerged or disappeared when naïve T cells undergo differentiation. The authors then compared the ChARs of T_EX_ cells post-chronic infection with those of effector/memory T cells post-acute infection. T_EX_ cells exhibit higher peak intensity of ChARs adjacent to exhaustion-associated genes (e.g., *Pdcd1*, *Havcr2*, *Batf*) than do effector/memory T cells. After examining the ChARs adjacent to *Pdcd1*, the authors indeed identified a new enhancer that promotes PD-1 expression in exhausted T cells (64).

Pauken et al. demonstrated the high stability of exhaustion-associated ChARs in CD8^+^ T_EX_ cells using an infection model. After establishing chronic infection and T cell exhaustion with LCMV-Cl13, Paueken et al. reinvigorated T cell response and cleared LCMV-Cl13 infection with PD-L1 blockade. Interestingly, after PD-L1 blockade and LCMV-Cl13 clearance, reinvigorated T cells fail to become functional memory T cells. The authors found that upon LCMV-Cl13 infection, antigen-specific T cells acquire an exhaustion-associated epigenetic profile distinct from those of effector and memory T cells. Although PD-L1 blockade reinvigorates T cell response against LCMV-Cl13, it largely fails to remodel the exhaustion-associated epigenetic profile. The authors conclude that epigenetic stability of T_EX_ cells limits durability of PD-L1–blockade effects (65).

Weber et al. showed that epigenetic remodeling occurs in exhausted chimeric antigen receptor (CAR)-T cells after transient rest. Rest is induced in CAR-T cells by using a drug-regulatable degron system. Rest for as few as four days induces global epigenetic remodeling in exhausted CAR-T cells. The remodeled epigenetic profile resembles that of the non-exhausted control CAR-T cells. Importantly, transient rest restores antitumor functionality in exhausted CAR-T cells (66). Taken together, T_EX_ cells acquire an epigenetic profile distinct from those of effector and memory T cells (**Figure 1**). The stability of exhaustion-associated epigenetic state may be different in exhausted CAR-T cells than in T_EX_ cells.

## Impact of the Tissue Environment and Metabolism

Anatomical location and proximity to antigen inform T cell states. This was noted early in chronic viral infection studies, where the more terminally differentiated T_EX_ cells resided in the red pulp of the spleen, the major site of LCMV infection (30). The less-differentiated T_PEX_ cells (retaining CXCR5 and TCF1) primarily localized to the T-cell zones of the white pulp (30). A recent study of type 1 diabetes showed that self-reactive CD8^+^ T cells in lymphoid tissue are less differentiated, retaining a hybrid of stem/naive and effector epigenetic programs. After infiltrating into pancreas, the site of the antigen, self-reactive CD8^+^ T cells exhibit mainly effector epigenetic programs and lose the stem/naive-like state (67).

Is the environment instructing T cell states, *via* ligand binding (30), nutrient availability (68–71), and local cytokine production (44)? Is antigen abundance (44, 72–74) due to proximity (30, 67) causing excess TCR stimulation (75), to push into terminal effector differentiation or exhaustion? The answers to these questions are far from clear and simple. As one example, CXCR5 is the major B cell zone chemokine receptor. How are CXCR5^+^ CD8^+^ T_PEX_ cells retained in the T cell zones (30)? CXCR5^+^ CD8^+^ T_PEX_ cells have high expression levels of *Xcl1*, which encodes a chemokine and promotes XCR1^+^ lymphoid dendritic cell interactions (30), present mainly in white pulp. This and other features of T_PEX_ cells (expressing CCR7 and CD69) may contribute to their retention in the T cell zones (76).

Defining the tissue-specific triggers of various T cell states will unlock our understanding of the pathogenicity of many immune-mediated conditions. As an example, environmental factors have long been presumed to play an essential role in triggering autoimmune diseases. For instance, high salt diet can trigger enhanced T follicular helper cells (Tfh) in systemic lupus erythematosus (SLE), a T cell compartment implicated in SLE pathogenesis, by inducing DNA methylation per recruitment of the hydroxytransferase Ten-Eleven Translocation 2 (TET2) (69). High NaCl also activates the p38/MAPK pathway in experimental autoimmune encephalomyelitis (EAE), enhancing another pathogenic T cell compartment, Th17 (70). Another study examined metabolic impacts on T cell function in leukemia treatment. Uhl et al. found that leukemic-cell-generated lactic acid impairs T cell glycolysis and proliferation. Mechanistically, lactic acid decreases the expression of glycolysis-related enzymes in T cells by lowering intracellular pH. Sodium bicarbonate (NaBi) treatment reverses the lactic acid-induced low intracellular pH and enhances graft-*versus*-leukemia activity of T cells (77). The tissue environment may represent an attractive target for improving or dampening T cell function. Likewise, Zhang et al. found that high glucose intake worsens EAE and colitis, upregulating mitochondrial ROS to mediate increased TGF-β production, thus promoting Th17 cell differentiation. Interestingly, T cell metabolism remained unchanged despite high glucose intake (78). Further studies examining metabolic drivers of T cell states are warranted.

How the local tissue microenvironment influences autoimmunity remains largely unknown. As mentioned above, self-reactive T cells are less differentiated in periphery and more terminally differentiated at target tissue sites (67). Chen et al. recently investigated the states of renal infiltrating T cells in lupus nephritis. Renal infiltrating T cells express PD-1 but most are PD-1^low^. Heightened renal hypoxia in lupus nephritis drives CD4^+^ and CD8^+^ infiltrating T cells to express hypoxia-inducible factor 1 (HIF-1), activating a HIF-1-PDK2-*bnip3* hypoxia survival axis. HIF-1 also overrides c-Myc, sponsoring glycolysis *via* NAD^+^-regenerating proline metabolism. HIF-1 blockade inhibits infiltrating T cells and reverses tissue hypoxia and renal injury. The authors thus suggest that chronic antigen stimulation in the kidney does drive the upregulation of PD-1 and exhaustion program in the PD-1^hi^ infiltrating T cells, yet despite this, many T cells retain pathogenic effector functions while still remaining PD-1^lo^ (79). Hypoxia-driven HIF circuits maintain T cell function in targeted tissues, even with chronic antigen stimulation and inhibitory receptors (79, 80).

As another example, in the tumor microenvironment, elevated extracellular potassium from cancer cell necrosis inhibits TCR-driven Akt-mTOR phosphorylation and TIL effector function. This inhibition is dependent on the activity of the serine/threonine phosphatase PP2A (81). Moreover, high levels of extracellular potassium modify the epigenetic program by a nutrient-deprived state (82). When nutrient uptake is impaired, nucleocytosolic acetyl-coenzyme A and methionine intermediate deficiencies lead to reduced histone acetylation of the effector program and maintenance of stemness (82).

Other local immune cells modify the tumor microenvironment and thus the T cell response. Intriguingly, tumor-associated macrophages can secrete endogenous glucocorticoids, which downregulate effector function and contribute to T cell dysfunction and poor checkpoint blockade response (68). M2-like suppressive macrophages within the tumor micro-environment form bidirectional inhibitory interactions with exhausted EOMES^hi^ CD8^+^ T cells, predicting worse outcomes (83).

Understanding the impact of tissue environment on T cell function, metabolism, and state-switching will enlighten our paradigms of transplant, infection, autoimmunity, and cancer.

## A Stepwise Model of Acquiring T Cell Exhaustion While Losing Stemness

Systems immunology approaches excel in revealing T cell states. Azizi et al. analyzed scRNA and TCR sequencing data from breast cancer. They showed that most T cells advance on continuous differentiation trajectories (84). Li et al. analyzed scRNA-seq data from melanoma. They indicate early effector CD8^+^ TILs transition into dysfunctional T cells in continuous progression (85). Based on the trajectory and TCR analysis of TILs in liver cancer, Zheng et al. showed that *GZMK* and *GZMA* expressing TILs represent an intermediate phase prior to terminal differentiation into T_EX_ cells (21).

Recently, Galletti et al. discovered two distinct stem/memory-like T cells differentiated by Granzyme K (GZMK) expression (86). The T cells retaining GZMK expressed an exhausted-like phenotype (T_PEX_) characterized by PD-1 and TIGIT inhibitory receptors, along with a transcriptional exhaustion signature (TOX, NFATC2, BATF, and others). In contrast, the T cells lacking GZMK showed a stem-like phenotype (T_SCM_), with expanded proliferative and cytokine-producing capacity. Interestingly, the GZMK^+^ T_PEX_ cells generated cytokines most effectively in a TCR-independent manner. The T cell receptor repertoire did not overlap between GZMK^+^ or GZMK^−^ populations, suggesting independence and potentially unique antigen-induction (86). Also, these T cell compartments appear among the normal human memory T cell population (86, 87), whereas most prior studies have focused on their existence in pathological states.

Beltra et al. described a stepwise model of progressive exhaustion, with four distinct transition states, differentiated with Ly108/TCF1 and CD69 (88). Two exhaustion progenitor states displayed similar chromatin signatures while maintaining distinct transcriptional and functional identities. T_EX_ progenitor one, quiescent and resident, transition to T_EX_ progenitor two, triggered by location change (from tissue to circulation-accessible) and proliferation. Next, a T_EX_ intermediate could arise, eventually becoming terminally differentiated (T_EX_ terminal). Transcription factors are dynamically expressed throughout the transition stages until reaching terminal differentiation, characterized by low levels of TCF1 and T-bet and high upregulation of TOX and Eomes. T-bet provoked the loss of TCF1. Future work could address if this loop feeds forward (88). TOX apparently antagonized T-bet, ultimately maintaining the upper hand as T-bet expression falls in T_EX_ terminal cells (88). T-bet also repressed PD-1 in T_EX_ cells, and PD-1 expression slowly faded as the T_EX_ cells progress to terminal differentiation (89). PD-1 may protect T_EX_ progenitors, through attenuation of TCR signaling (30, 36, 88).

Taken together, the understanding of T cell states during chronic antigen exposure has exploded in the past years. From the TCF1^+^ T_PEX_-to-TCF1^–^ T_EX_ transition model of exhaustion, a more sophisticated, stepwise model of T cell state-switching has evolved.

## Perspectives on T Cell Stemness and Exhaustion in Organ Transplantation

Solid organ transplantation is a lifesaving treatment for patients with end-stage organ failure. Allogeneic T cell response plays a decisive role in rejecting the transplanted organs. Most transplant patients must take immunosuppressive drugs daily to inhibit allogeneic T cell response as long as the transplanted organs remain functional. Yet, immune rejection and immunosuppressive drug side effects still limit long-term allograft survival (90).

A question lingers: why does daily immunosuppression often fail to eliminate allogenic T cell response completely? Do TCF1^+^ stem-like T cells enable the persistence of allogeneic T cell response? Lipson et al. reported a case in which an anti-PD-1 antibody was used to treat metastatic cancer in a kidney transplant patient. Though the anti-PD-1 treatment invigorated antitumor response, it induced transplant rejection (91). Later, Murakami et al. reported that among 69 kidney transplant patients who received immune checkpoint blockades to treat malignancies, 29 of whom developed acute rejection and 19 of whom lost their kidney allografts (92). It remains unknown whether immune checkpoint blockades invigorate TCF1^+^ stem-like allogenic T cells in key transplant patients.

Although few data have been reported to date, higher levels of circulating T_EX_ cells have been shown to be associated with improved transplant outcome. Levitsky et al. observed increases in T_EX_ cell populations in patients with tolerant liver transplants (93). Fribourg et al. identified both CD4^+^ and CD8^+^ circulating T_EX_ cells in kidney transplant patients. Increase of T_EX_ cell frequency after lymphocyte-depleting induction therapy correlates with improved transplant function (94).

We have previously found that deletion of a transcription factor IRF4 in T cells leads to transplant tolerance. Mechanistically, allogeneic *Irf4*-deficient CD4^+^ T cells progressively develop into an exhaustion-like phenotype, evident by their high PD-1 expression and impaired cytokine production (95). Miller et al. used CD154 blockade plus donor-specific transfusion to induce transplant tolerance. This tolerogenic protocol also induces a PD-1^hi^ phenotype of alloreactive T cells (96). Both IRF4 deletion and CD154 blockade eliminate T cell effector function. The T cell state under those tolerogenic conditions was thus named “T cell dysfunction” rather than exhaustion.

To investigate whether T cell exhaustion naturally occurs in transplantation without immunosuppression or gene deletion in T cells, we transplanted female B6 recipients with either large-size whole-tail skins or small-size tail skins from male B6 donors. Large- but not small-size male skins induce exhaustion of anti-male CD8^+^ cells and are subsequently accepted by female recipients. In another model, we studied the natural exhaustion of allogenic CD4^+^ T cells. We used TCR-transgenic B6 TEa CD4^+^ cells that recognize Balb/c I-Eα antigen. TEa CD4^+^ cells were adoptively transferred either into B6 recipients that received Balb/c skins or into F1 offspring of B6 and Balb/c mice (CB6F1) that contain abundant I-Eα antigen. TEa cells in B6 recipient mice responded to Balb/c skin transplantation and were not exhausted. By contrast, almost all TEa cells were quickly exhausted after adoptively transferring into CB6F1 mice. TEa cells in CB6F1 mice expressed high levels of NFATc1, TOX, NR4A1, and NR4A2. Thus, the abundant I-Eα antigen in CB6F1 mice may drive the TCR-NFAT-TOX/NR4A exhaustion program in TEa cells (44).

Results from our exhaustion models conclude that high antigen load induces allogeneic T cell exhaustion. This may help explain why large liver allografts achieve tolerance more often than other organ allografts (93). This may also clarify why lymphocyte-depleting induction therapy induces allogeneic T cell exhaustion, as it dramatically increases the antigen-to-T cell ratio (94). Currently, transplant recipients require lifelong use of FK506 or other immunosuppressive drugs. Although FK506 very effectively inhibits T cell effector programs, it also prevents T cell exhaustion by inhibiting the NFAT-TOX/NR4A axis (45, 47, 53, 97). For effective induction of transplant tolerance, it is essential to identify therapeutic approaches that not only inhibit the effector programs, but also promote allogeneic T cell exhaustion. Moreover, the role of T cell stemness in transplantation remains largely unknown and needs to be explored.

## Conclusions

Nuanced T cell state dysregulation underpins the induction and maintenance of many disease states, notably organ transplant rejection, autoimmunity, chronic infection, and cancer. The past few decades have yielded tremendous advances in our understanding of transcriptional, epigenetic, and metabolic regulations of T cell stemness and exhaustion. Reinvigoration of T cells in the tumor microenvironment with checkpoint blockade, for example, demonstrates the therapeutic power gained from understanding T cell states. T_PEX_ exhaustion precursors can self-renew, give rise to terminally differentiated T_EX_ cells, and respond to checkpoint blockade with a proliferative burst. C-Myb, Wnt, and TOX orchestrate TCF1 expression and thereby influence exhaustion or effector fate. Similarly, BACH2 inhibits AP-1 to sustain T_PEX_ cells, while BATF and NR4A1 inhibit AP-1 to drive terminal exhaustion. T_PEX_ cells and T_EX_ cells have distinct epigenetic signatures, impacted by resting states and hypoxia, to name a few. Antigen load also drives the exhaustion program, potentially *via* the TCR-NFAT-TOX/NR4A axis.

These exciting new discoveries of various T cell states and their regulators may give us the insight to target or exploit transition points. Currently, the mainstay of immunosuppressive therapies primarily target effector cells, while the quiescent T_PEX_ cells can persist unperturbed, maintaining disease. Finding ways to modulate T_PEX_ and T_EX_ cell fate and function could revolutionize treatment in transplant rejection and autoimmunity.

As more mechanistic subtleties are brought to light, we can eventually learn to not merely harness the destructive power of the immune system, but also to tame it.

## Author Contributions

All authors listed have made a substantial, direct, and intellectual contribution to the work, and approved it for publication.

## Funding

This study was supported by the US National Institutes of Health grant (#NIH R01AI132492 to WC).

## Conflict of Interest

The authors declare that the research was conducted in the absence of any commercial or financial relationships that could be construed as a potential conflict of interest.

## Publisher’s Note

All claims expressed in this article are solely those of the authors and do not necessarily represent those of their affiliated organizations, or those of the publisher, the editors and the reviewers. Any product that may be evaluated in this article, or claim that may be made by its manufacturer, is not guaranteed or endorsed by the publisher.

## Supplementary Material

The Supplementary Material for this article can be found online at: https://www.frontiersin.org/articles/10.3389/fimmu.2021.725618/full#supplementary-material
