# Supplementary material for: Schrödinger’s T Cells: Molecular Insights Into Stemness and Exhaustion
Source: Front Immunol. 2021 Aug 26;12:725618. doi: 10.3389/fimmu.2021.725618 (PMC8427607; doi:10.3389/fimmu.2021.725618)
Supplement: Supplementary file 1 [file Table_1.docx]

**Supplementary Table:** abbreviations used throughout the manuscript

| Abbreviation | Definition |
| --- | --- |
| ADA | adenosine deaminase |
| Akt | serine/threonine protein kinase |
| AP-1 | activator protein 1 |
| BACH2 | broad complex-tramtrack-bric a brac and Cap'n'collar homology 2 |
| BATF | basic leucine zipper ATF-like transcription factor |
| Bcl-2 | B cell lymphoma 2 |
| *Bnip3* | BCL2 interacting protein 3 (gene encoding Bnip3) |
| CAR-T | chimeric antigen receptor T cell |
| CB6F1 | F1 offspring of B6 and Balb/c mice |
| ChAR | chromatin accessible region |
| ChIP-seq | chromatin immunoprecipitation-sequencing |
| c-Jun | cellular Jun (AP-1 transcription factor subunit) |
| c-Myb | cellular Myb (MYB proto-oncogene, transcription factor) |
| CNS | central nervous system |
| CXCR3 | C-X-C motif chemokine receptor 3 |
| CXCR5 | C-X-C motif chemokine receptor 5 |
| *Dnmt3a* | DNA methyltransferase 3A (gene encoding DNMT3A) |
| DTR | diphtheria toxin receptor |
| EAE | experimental autoimmune encephalomyelitis |
| *Entpd1* | ectonucleoside triphosphate diphosphohydrolase-1 (gene encoding CD39) |
| EZH2 | enhancer of zeste homolog 2 |
| GZMA | granzyme A |
| GZMK | granzyme K |
| H3K9 | histone 3 lysine 9 |
| H3K9me3 | histone 3 lysine 9 trimethylation |
| H3K27 | histone 3 lysine 27 |
| H3K27me3 | histone 3 lysine 27 trimethylation |
| *Havcr2* | hepatitis A virus cellular receptor 2 (gene encoding TIM3) |
| HIF-1 | hypoxia-inducible factor 1 |
| HSCT | hematopoietic stem cell transplantation |
| Id3 | inhibitor of DNA binding 3 |
| IE-α | MHC class II I-Eα subunit |
| IFN-γ | interferon gamma |
| IL-2Rα | interleukin-2 receptor subunit alpha |
| IL-2Rβ | interleukin-2 receptor subunit beta |
| IL-7Rα | interleukin-7 receptor subunit alpha |
| IRF4 | interferon regulatory factor 4 |
| LAG-3 | lymphocyte activation gene 3 |
| LCMV | lymphocytic choriomeningitis virus |
| LCMV-Cl13 | LCMV clone 13 |
| LCMV-D | LCMV strain Docile |
| KLRG1 | killer cell lectin-like receptor G1 |
| mTOR | mechanistic target of rapamycin |
| MAPK | mitogen-activated protein kinase |
| NaBi | sodium bicarbonate |
| NFAT | nuclear factor of activated T cells |
| NFATC2 | nuclear factor of activated T cells 2 |
| NR4A | nuclear receptor subfamily 4A |
| PD-1 | programmed cell death protein 1 |
| *Pdcd1* | programmed cell death protein 1 (gene encoding PD-1) |
| PD-L1 | programmed cell death - ligand 1 |
| PDK2 | pyruvate dehydrogenase kinase 2 |
| PRC2 | polycomb repressive complex 2 |
| ROS | reactive oxygen species |
| Sca-1 | stem cell antigen-1 |
| scRNA-seq | single cell RNA sequencing |
| SLE | systemic lupus erythematosus |
| SUV39H1 | suppressor of variegation 3-9 homolog 1 |
| TCF1 | T cell factor 1 |
| *Tcf7* | transcription factor 7 (gene encoding TCF1) |
| T_CM_ | central memory T cell |
| TCR | T cell receptor |
| T_EFF_ | effector T cell |
| T_EM_ | effector memory T cell |
| T_EMRA_ | terminally differentiated memory T cell |
| TET2 | Ten-Eleven Translocation 2 |
| T_EX_ | exhausted T cell |
| Tfh | T follicular helper cell |
| TGF-β | transforming growth factor beta |
| Th17 | T helper 17 cell |
| TIGIT | T cell immunoreceptor with Ig and ITIM domains |
| TIL | tumor-infiltrating lymphocyte |
| TIM-3 | T cell immunoglobulin and mucin-domain containing protein-3 |
| TK | thymidine kinase |
| TNF-α | tumor necrosis factor alpha |
| T_PEX_ | precursors of exhausted T cell |
| T_SCM_ | memory stem T cell |
| TOX | thymocyte selection associated high mobility group box |
| YF | yellow fever |
| *Xcl1* | X-C motif chemokine ligand 1 (gene encoding XCL1) |
| XCR1 | X-C motif chemokine receptor 1 |
| *Zeb2* | zinc finger E-box binding homeobox 2 (gene encoding Zeb2) |
